# Supplementary material for: Opposing neural processing modes alternate rhythmically during sustained auditory attention
Source: Commun Biol. 2024 Sep 12;7:1125. doi: 10.1038/s42003-024-06834-x (PMC11393317; doi:10.1038/s42003-024-06834-x)
Supplement: Supplementary file 3 — Description of Additional Supplementary Materials [file 42003_2024_6834_MOESM3_ESM.pdf]

Description of Additional Supplementary Files

File name: Supplementary Data

Description: Source data behind graphs in the paper
